# Comparison of Milled Full-Arch Implant-Supported Frameworks Realised with a Full Digital Workflow or from Conventional Impression: A Clinical Study

**DOI:** 10.3390/ma16020833

**Published:** 2023-01-15

**Authors:** Francesco Pera, Paolo Pesce, Francesco Bagnasco, Nicolò Pancini, Massimo Carossa, Lorenzo Baldelli, Marco Annunziata, Marco Migliorati, Domenico Baldi, Maria Menini

**Affiliations:** 1 C.I.R. Dental School, Department of Surgical Sciences, University of Turin, 10126 Turin, Italy; 2 Department of Surgical Sciences (DISC), University of Genoa, 16132 Genoa, Italy; 3 Multidisciplinary Department of Medical-Surgical and Dental Specialties, University of Campania “Luigi Vanvitelli”, 81100 Caserta, Italy

**Keywords:** dental implants, digital impression, intraoral scanner, full-arch, accuracy

## Abstract

Background: The aim of the present study was to investigate the accuracy of a new digital impression system, comparing it to the plaster impression technique in the realization of full-arch implant-supported metal frameworks. Methods: We took 11 scans (8 of the upper maxilla and 3 of the lower jaw) on a sample of nine patients previously rehabilitated with fixed full-arch screw-retained prostheses following the Columbus Bridge Protocol (CBP) with four to six implants (total: 51) since at least 4 months. Two impressions were taken for each dental arch: one analogic plaster impression using pick-up copings and an open tray technique and a second one using an intra-oral scanner. Two milled metal substructures were realised. The precision and passivity of the substructures were clinically analysed through the Sheffield test and endo-oral radiographs. Laboratory scans of the plaster casts obtained from an intra-oral scanner (IOS) and of the plaster casts obtained from traditional impression were compared with the intraoral scans following Hausdorff’s method and an industrial digital method of optical detection to measure discrepancies. A Mann–Whitney test was performed in order to investigate average distances between surfaces after the superposition. Results: The Sheffield test demonstrated an excellent passivity of the frameworks obtained through both the digital and the analogic method. In 81.81% of cases (n = 9) both substructures were found to have a perfect fit with excellent passivity, while in 18.18% (n = 2) of cases the substructures were found to have a very slight discrepancy. From the radiographic examination, no gaps between the frameworks and the implant heads or multiunit abutments were observed, with 100% accuracy. By superimposing digital files of scans according to Hausdorff’s method, a statistically significant discrepancy (*p* = 0.006) was found between the digital scans and the digital models obtained from plaster impressions. Three-dimensional optical detection found a mean discrepancy of 0.11 mm between the analogic cast and the cast derived from the digital impression. Conclusions: The present study clinically demonstrates that milled implant-supported full-arch frameworks obtained through a digital scan and the herein described technique have an accuracy comparable to those obtained with traditional plaster impression.

## 1. Introduction

Computer-aided design and manufacturing (CAD-CAM) allows the obtainment of a three-dimensional object starting from a digitally performed vector drawing [1], and over the last forty years, it has revolutionised the production world, including prosthodontic workflows.

The idea of using CAD-CAM techniques for dental restorations was first conceived in 1983 by Francois Duret, [2] who created the first CAD-CAM dental artifact in 1983. In 1985, Morman and Brandestini realised an inlay for the first time using the first optical scanning system coupled with a milling device (CEREC: CEramic REConstruction) [3,4].

The advent of CAD-CAM marked history in the dental community, and since then advances in modern scanning techniques have brought this technology to a broad applicability. In particular, over the last two decades, much research has led to the success of dental CAD-CAM technology and multiple methods have been proposed to collect three-dimensional data of teeth and implants through intraoral optical cameras and laser scans.

Making an accurate dental impression is a crucial step in any implant treatment [5].

Inaccurate transfer of implant position can lead to inadequate prosthodontic fit, which can strain the prosthodontic components and possibly lead to complications [6].

Impression procedures on implants traditionally involve the use of impression copings or transfers [7]. However, using modern technology, it is now possible to eliminate this step and follow digital workflows for the creation of implant-supported prostheses [8].

The digital workflow can be direct or indirect. Indirect digitalization provides a scan of the master cast or the analogic impression using a laboratory scanner [9].

Direct digital workflows, instead, include the use of intraoral scan bodies (ISB) and intraoral scanners (IOS) to directly generate digital scans from the patient’s mouth [9].

IOSs collect information about the shape and size of the dental arches (and the position of dental implants) through the emission of a light beam. The information collected is processed by a software, which, through dedicated algorithms, accurately reconstructs the 3D virtual model of the desired structures. The scanner first creates a “point cloud” and from this derives a polygonal grid called a wire-frame (mesh density). The latter is created by the computer algorithm, through which the points of the cloud are joined. The scan is finally reworked to obtain the final 3D model in STL format [10].

However, these scanning systems are inadequate for detecting pure geometric shapes, such as engagements and planes (i.e., trefoil hexagons, conometry) that are today common in dental implants [11].

To manage this clash between implant geometry and scanning, many systems combining optical scanning with probing scans have been proposed. In single-unit and short-span rehabilitations, IOSs using ISB have shown similar accuracy to conventional impressions [12].

However, digital impression of edentulous dental arches is still considered a challenge today and shows less accuracy compared to partial rehabilitations in studies published in past years. In addition, accuracy differs, with some IOSs performing better than others [13].

Although the accuracy of digital impressions in full-arch rehabilitations is yet to be verified in vivo, lately, several in vitro studies have shown that digital impressions have an accuracy that is very close to impressions made with plaster, which, however, still remains the gold standard [14,15,16,17,18].

Although the overall quality of the data is highly dependent on the specific IOS system, the surface topography and characteristics of the surfaces to be scanned can affect the accuracy of the digital impression, causing less accuracy if less texture is present [19].

When attempting to scan an edentulous arch using ISB, one of the main difficulties is due to the small number of fixed reference points, the distance between the scan bodies, and the fact that this distance with a small number of reference points cannot guarantee satisfactory scan quality [20].

When the reference points are limited, the images may not be stitched together properly, or parts of the scan may result in redundant data [21].

Therefore, techniques have been proposed to overcome this issue by increasing the number of reference points in an edentulous arch by modifying the surface and topography of the edentulous ridge [22]. Different tissue additives, powders, and adhesive radiopaque markers; different amounts of pressure indicator paste (PIP); and spray and resin markers made in composite resin have been suggested [23].

Few published studies have clinically evaluated IOS in full-arch implant-supported rehabilitations [24], and to the authors’ knowledge, this is the first clinical study comparing frameworks realised with both techniques. Therefore, the aim of the present clinical trial was to investigate the trueness of a new IOS in completely edentulous patients rehabilitated with four to six implants per dental arch.

In particular, the aim was to compare the accuracy of milled metal frameworks realised on the base of a new IOS, with those realised using the plaster impression technique already validated in many years of clinical use for the manufacturing of full-arch implant-supported frameworks [25,26,27].

The null hypothesis tested was that there were no differences in accuracy between the two methods applied.

## 2. Materials and Methods

The protocol was approved by the local ethical committee of the University of Genoa (CERA). The study protocol was carefully described to the patients, and they signed an informed consent before starting the investigation. Patients were recruited for the present clinical study at the Division of Prosthodontics and Implant Prosthodontics of the University of Genoa (Department of Surgical Sciences, DISC). The patients were regular patients of the division and had to be previously rehabilitated with immediate loading full-arch rehabilitations following the Columbus Bridge Protocol (CBP) [26,28,29,30,31] at the same division since at least 4 months, with 4–6 osseointegrated implants and fully healed peri-implant tissues.

The Columbus Bridge Protocol is a surgical–prosthodontic protocol that allows the restoration of aesthetics, phonetics, and function in patients with severely compromised teeth in 48 to 72 h. It provides the placement of implants with a minimum length of 10 mm in native bone with a high insertion torque, placed upright in the anterior regions of the jaws and inclined in the posterior regions to avoid noble anatomical structures. Angled multiunit abutments are used to correct implant inclination. A fixed screw-retained prosthesis is delivered within 48 h after surgery, with a rigid metal framework, no extension, and composite resin veneering material. The week before impression, a professional oral hygiene session is planned. Then, the day of the impression, the screw-retained full-arch fixed prostheses were removed, and all the implants were carefully checked to ensure their stability. Multiunit abutments were tightened to verify that they were not loosened. All the patients had external hexagon implants.

For each dental arch, two impressions were taken: one traditional impression using impression plaster (Snow White plaster, Kerr) and an open tray technique with pick-up copings, and a second impression using a new IOS (Mach2 Intraoral Scanner Shining 3D, distributed by Euromax Monaco). Mach2 uses colour photogrammetry technology, with a scanning speed of 15 frames per second (video) and a scanning field of 11 × 11 mm. The manufacturer does not recommend powders to opacify the elements to be scanned and the output file is an open STL format.

**Figure 1:**
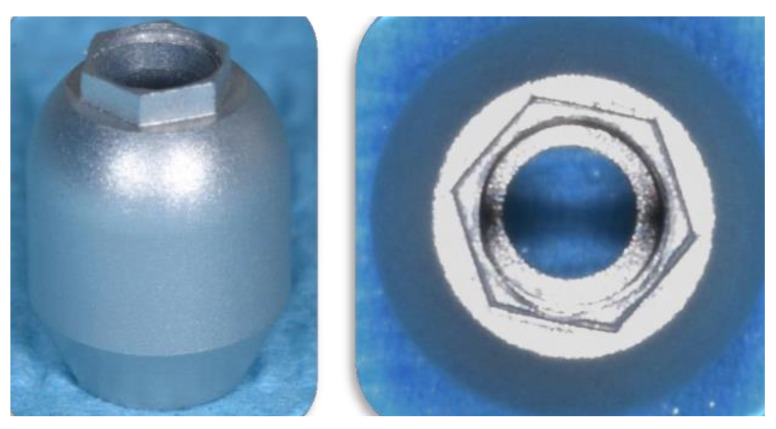
Scan body for edentulous arches to be screwed directly on the implant head.

The scan-bodies used were specifically designed for edentulous patients (Toothless^®^ Scanbody, Mech & Human, Grisignano di Zocco, Italy). They had a rounded shape with a hexagon on the top, useful to increase the reference points for the operation of best fitting. Scan-bodies were available both to be screwed directly on implant heads or on MUAs. They were identical except for their connection (Figure 1) [18].

One experienced operator (FP) made all the digital and analogic impressions. The digital impression was performed using an “S” scan path; the scanner tip followed the entire arch with a fluid movement starting from the most distal implant of the first quadrant to the contralateral implant while zig-zagging from the vestibular to the palatal side and back. Once the scans were taken, after placing the pick-up transfers, the plaster impression was taken. The plaster impression was sent to the dental laboratory to immediately pour it and obtain a master model. The stl file obtained with the IOS was also directly sent to the dental laboratory. In the following 48 h, two cobalt chromium substructures were milled for each dental arch: one on the base of the digital impression and one on the base of the analogic plaster impression. All the frameworks were fabricated with the implant cylinders luted to the metal framework [32].

The frameworks were performed by two different technicians depending on their experience and specialization:

- The first dental technician poured the conventional impression, obtaining a plaster master model that was scanned with a laboratory scanner. The digital file obtained was used to mill the framework [33,34],
- The second technician realised the framework on the base of the digital impression.

### 2.1. Clinical Evaluation

All substructures were screwed onto the patient and evaluated by two clinicians.

Precision and passivity of the substructures were clinically analysed through the following methods:

- Sheffield Test: The framework was considered passive fitting when tightening one screw on the distal abutment (tightening torque: 10 Ncm with a dynamometric screwdriver) did not create a gap at the other framework-implant interfaces, as detected using magnification devices (Zeiss 4x). If the fit was not sufficient, the superstructure lifted when the contralateral screw was tightened, creating a gap at the level of one or more abutments. After this examination, all the prosthetic screws were tightened and the clinician recorded if he felt a possible feeling of strain while manually tightening. The fit of each single framework as evaluated through the Sheffield test was categorised according to the following classification arbitrarily defined by the authors:
  3 or excellent: No strain is perceived while screwing the framework. At the visual examination, no detachment can be detected between the framework and the implant head/MUA while tightening the contralateral screw.
  2 or good: A strain is perceived while screwing the framework, but the framework can be seated in place manually while tightening the screw and/or upon visual examination while tightening the contralateral screw; a slight detachment of less than 1 mm is detected between the framework and the MUA or the implant head; no detachment can be observed after screwing.
  1 or very bad: The prosthesis strains and it is not possible to position it in its seat and/or upon visual inspection; before tightening, there is a gap between the framework and the MUA/implant ≥1 mm and/or a detachment is detected between the framework and the MUA or the implant head also after screwing.
- Endo-oral radiographs: The evaluation of the precision of the metal framework was also evaluated with intraoral digital periapical radiographs taken with the parallel technique after tightening all the prosthetic screws. Intraoral radiographs were performed in each patient both with the framework obtained using the classical method and the framework obtained via IOS. A digital software (OrisWin DG, FONA- Dental, Assago, Italy) was used to perform measurements. The software was calibrated for every image using the implant diameter as reference. The passivity of the framework according to intraoral radiographs was defined on the basis of the following parameters:
  1 or excellent: if the framework is found to be seated in place without any gap at the interface with the MUA/implant head,
  2 or bad: if the framework is not seated in place and presents gaps at the interface with the MUA/implant head.
  Two of the authors (NP and FB) performed the radiographic evaluations on the mesial and distal surfaces of each implant.

### 2.2. Digital Laboratory Analysis

In both the digital and the analogic procedure, a plaster cast containing the implant analogs was made so that two plaster casts were realised for each dental arch:

- Cast 1: realised on the base of the plaster impression, the cast was made immediately after the plaster impression was taken.
- Cast 2 (reverse cast): realised on the base of the digital impression, a prototype prosthesis containing the luting cylinders was produced and then, after coupling them onto analogic analogs, a plaster cast was manufactured.

**Figure 2:**
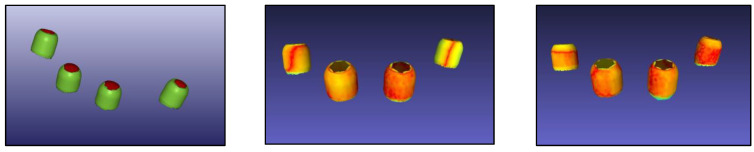
(**Left**—intraoral scan; **Center**—superimposition of the intraoral scan with the scan of cast 1; **Right**—superimposition of the intraoral scan with the scan of cast 2.

The laboratory analysis of this study carried out with digital technology aimed to measure the differences between the two casts. The detection of the positions of the implant analogues in the casts was carried out in two different ways:

(a) with a digital method, comparing the intraoral scans with the scans of the two plaster models. The casts were scanned with a laboratory extraoral scanner, Shining 3d (Mech&Human), providing a standard resolution of 25 to 50 μm and an average error of 5 to 10 μm. The stl files of cast 1 and 2 were superimposed to the intraoral scan to measure discrepancies in implant position using reverse engineering software MeshLab (http://meshlab.source-forge.net). The superimposition of files according to the Hausdorff method was used to measure discrepancies. Mean distances between surfaces were obtained (Figure 2) [35].
(b) with an industrial digital method of optical detection, as described below.

An optical 3D measurement technique was applied to measure discrepancies in analogues position using ATOS Q (GOM ZEISS), which offers triple scanning technology with a narrow-band blue light (light source: led) and presenting the following characteristics:

- Scan points: 8 million,
- Measured area: 100 × 70–500 × 370 mm²,
- Distance of the points: 0.04–0.15 mm,
- Working distance: 490 mm

Before starting each measurement, a calibration was carried out by analysing a reference sample of known dimensions. The parametric software GOM inspect Pro (GOM ZEISS Company) was used for the analysis. After superimposing the two files generated with the optical 3D measurement, a point was chosen for each superimposed implant position and a cumulative comparison for the 3 cartesian axes (X, Y, Z), that is, the distance between points, was measured in mm. For the same points, the discrepancies were also measured for each cartesian axis (X, Y, Z).

### 2.3. Statistical Analysis

A descriptive analysis was carried out on the calculated mean values of the measured distances between the points. A Mann–Whitney test was performed in order to investigate average distances between surfaces after the superposition of files according to the Hausdorff method.

*p* < 0.05 was considered statistically significant and SPSS Statistics (Statistical Package for Social Science, v.21, IBM) was used for the computation.

## 3. Results

Nine patients (four women and five men; mean age: 71.6 years, range: 44–87) were included in the present research.

Six patients were treated at the upper jaw and one at the lower jaw, and two presented a CBP rehabilitation in both arches, so that eleven dental arches were included in the research (eight maxillae and three mandibles). Seven dental arches were rehabilitated with four implants, one with five implants and three with six implants, for a total of fifty-one dental implants.

Thirty-nine implants presented multiunit abutments (MUA) to correct their inclination, while twelve implants were rehabilitated directly on the implant head.

The Sheffield test demonstrated optimal outcomes for both the techniques investigated. In 81.81% of dental arches (n = 9), both substructures revealed an excellent passivity, and only only substructures, one derived from the digital impression and one derived from the traditional impression, presented a passivity that was categorised as “good”.

From the radiographic examination, no gaps between the frameworks and the implant heads or MUAs were observed, resulting in a 100% accuracy.

The following table shows the mean values of discrepancies (distances) between the points measured with the Hausdorff method (Table 1). A statistically significant difference was present (*p* = 0.006) in the discrepancies between cast 1 and cast 2 and the digital scan, with lower discrepancies for cast 2.

The following table shows the mean values of discrepancies (distances) between the points measured with the optical 3D method (Table 2). Point 5 and point 6 were available only for four and three dental arches, respectively.

Rear points were those with the higher distance between the two models.

The mean distance by considering the full sample of 51 observations was +0.11 (Std. Dev. 0.06). Furthermore, discrepancies on the three axes X, Y and Z were computed for the three axes (Table 3).

## 4. Discussion

Based on the outcomes of the present research, the null hypothesis was only partially rejected. In fact, no differences were identified among the two techniques when the frameworks were checked clinically (Sheffield test and radiographic analysis). Similar clinical results suggest that both techniques could be successfully used in full-arch rehabilitations.

In contrast, a statistically significant difference was identified among casts obtained via digital and analogic impressions with lower discrepancies for digital ones when compared with the intraoral scan following the Hausdorff method. However, this evaluation is not free from bias. In fact, it might be considered predictable that cast 2 (derived from the digital impression) is more similar to the digital impression itself than the digitised conventional cast, with mean discrepancies of 130 µm for cast 1 and 80 µm for cast 2. On the base of the present study, it is not possible to determine in which phase the discrepancy originated. However, it is the authors’ opinion that it may have arisen during the laboratory procedure of cast realization.

Digital evaluation using an optical 3D measurement revealed a mean discrepancy in analogue positions in the two casts of 0.11 mm.

Clinical studies comparing digital and conventional impression techniques in full-arch rehabilitations are scarce. Similar to the present outcomes, Cappare et al. [36] found no voids at the framework–implant connection when evaluated through intraoral digital radiographs. However, clinical examination might not be sufficient to evaluate accuracy and different techniques have been proposed. Similar to the present research, some authors have evaluated discrepancies between the digitised conventional model and the intraoral scan.

Papaspyridakos et al., in a study analysing full-arch implant impressions obtained digitally or conventionally, reported a difference of 88 µm between the digitised conventional stone casts and the intraoral digital scans [24]. In the present research, a slightly greater discrepancy was found between digitised conventional casts and intraoral scans (130 µm). The differences might be due to the different clinical and laboratory protocols applied; however, in both cases, the discrepancy lies within the clinically acceptable limit.

Other authors reported greater discrepancies: the investigation by Chochlidakis et al. [37] reported a difference of 162 µm among the digitised conventional model and IOS. However, several differences in clinical and laboratory procedures, including the use of a different IOS, could have affected the outcomes.

Compared with the above-mentioned clinical studies, in the present research, a cross-over approach was chosen. Two frameworks were realised for each dental arch and clinically tested: one realised from the digital impression and one on the base of the conventional impression to reduce possible inter-patient variables.

Full-arch rehabilitations are reported in the literature to be the type of rehabilitation that commonly present a higher risk of misfits due to possible distortions during laboratory procedures [38]. For these reasons, many protocols have bypassed this problem by performing an in-mouth cementation of the prefabricated prosthesis, without a rigid substructure, on titanium abutments screwed directly onto the implants or onto the multiunit abutments.

This, however, is a risky choice since the framework is essential to achieve splinting and guarantee immobility of the implants and even load distribution, especially when the prosthetic volume is small [34,39,40].

Fundamental to successful implantology is the achievement of a passive fit of the prosthetic superstructure. Although it has not been clinically confirmed, a poor fit of the implant–prosthetic superstructure can lead to unfavourable complications [41,42]. These can range from fracture of various implant components, pain, loss of marginal bone, and even loss of osseointegration. Indeed, misfits are reported to be one of the possible variables that can affect the long-term success rate of the rehabilitations [43].

Therefore, minimising misfit and optimising passive fit is considered a prerequisite for the long-term survival and success of implants and prostheses and research on the topic remains actual.

A passive fit is achieved if it does not lead to static loads and forces on the prosthesis and peri-implant bone tissue. To define passive fit, factors such as mechanical or processing tolerance, i.e., the discrepancy between the various components when they are held in place by their respective fixation screws, must also be considered. This can be considered as a source of mismatch, which can range from 22 to 100 microns [44,45].

In the present study, both digital and analogic impressions led to excellent clinical fit when coupled with the laboratory luting technique in order to cement implant cylinders to milled frameworks, in order to compensate for possible distortions.

In the present clinical investigation, impressions were taken at healed implant sites. To date, the obtainment of accurate digital impressions is still not considered predictable immediately after implant insertion in full-arch rehabilitations due to the presence of blood and the reduced landmarks present during scanning. The present results suggest that the digital impression could be used on healed gingiva, making it a reliable tool for the manufacturing of the final prosthesis. Future studies must focus the attention on the use of IOS directly after implant insertion.

Some limits of the present research must be acknowledged. First of all, a power analysis was not performed; secondly, the frameworks were created by two different dental laboratories, and this might represent a source of bias. However, it should be underlined that each laboratory was considered an expert in the technique executed (digital or analogic). An additional limit could be the small sample of patients.

## 5. Conclusions

This is the first clinical study to investigate the accuracy of milled frameworks in patients treated with full-arch implant-supported rehabilitations obtained via digital scanning and plaster impressions.

Within the limitations of this study, digital scanning was found to produce frameworks of equal fit to those obtained through plaster impressions.

## Figures and Tables

**Figure 3:**
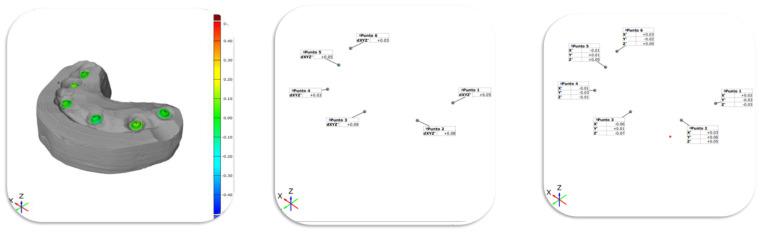
Points identified in the analysis.

**Table 1:** The table shows mean values of discrepancies measured with the Hausdorff method (mm).

	Discrepancy between Cast 2 and the Intraoral Scan	Discrepancy between Cast 1 and the Intraoral Scan
*p* = 0.006	0.08	0.13

**Table 2:** Mean values and standard deviations of discrepancies among the six points (as shown in Figure 3) evaluated with the optical 3D method (mm).

	Point 1 (n = 11)	Point 2 (n = 11)	Point 3 (n = 11)	Point 4 (n = 11)	Point 5 (n = 4)	Point 6 (n = 3)
Mean (SD)	0.13 (0.06)	0.09 (0.03)	0.10 (0.04)	0.16 (0.08)	0.12 (0.07)	0.06 (0.04)

**Table 3:** Mean cumulative discrepancies on the three axes X, Y, and Z.

	X	Y	Z
Mean Dist	+0.03	+0.01	+0.02
St. Dev	0.10	0.05	0.05

## Data Availability

Data are available on request.
